# Segmental hair analysis for flunitrazepam and 7-aminoflunitrazepam in users: a comparison to existing literature

**DOI:** 10.1080/20961790.2020.1824600

**Published:** 2020-10-22

**Authors:** Yue Zhuo, Ping Xiang, Jingjie Wu, Xin Wang

**Affiliations:** a Department of Forensic Medicine, Guizhou Medical University, Guiyang, China; b Department of Forensic Toxicology, Academy of Forensic Science, Shanghai Key Laboratory of Forensic Medicine, Shanghai, China; c Institute of Forensic Medicine, Guizhou Police College, Guiyang, China

**Keywords:** Forensic sciences, forensic toxicology, segmental hair analysis, flunitrazepam, 7-aminoflunitrazepam, liquid chromatography–tandem mass spectrometry

## Abstract

The availability of more quantitative data on flunitrazepam (FLU) and 7-aminoflunitrazepam (7AF) would aid in obtaining a better understanding of the interpretation of FLU concentrations in human hair. The purpose of this study was to provide concentrations of FLU and 7AF in hair segments of 22 FLU users. Quantitative data regarding hair concentrations of FLU and 7AF from various types of cases were also reviewed to give a comprehensive overview of the comparability of different studies. Three to six 1 cm segments of scalp hair from 22 FLU users were analyzed by a liquid chromatography–tandem mass spectrometry (LC–MS/MS) method. FLU and its metabolite were confirmed in the hair segments from all cases. Concentrations of FLU and 7AF in the segments ranged from 0.01–0.16 ng/mg (median of 0.03) and 0.01–0.34 ng/mg (median of 0.09), respectively. Most cases had FLU and 7AF distributions along the hair segments that were suggestive of repeated drug use. A summary of the published concentrations gives valuable data and can assist forensic investigators in their estimations of drug use history and patterns.

**Key points**
- A method using LC–MS/MS to quantify flunitrazepam and its metabolite was described.
- Segmental analysis of flunitrazepam and its metabolite in human hair was reported.
- A comprehensive overview of quantitative data was given.

## Introduction

Studying hair provides researchers with the advantage of a wide detection window, which can be particularly useful for the assessment of retrospective drug exposure in cases of drug abuse or drug-facilitated crimes (DFCs) and in postmortem toxicology [1–3]. The head hair grows approximately 1 cm per month, with a range of about 0.5–1.5 cm/month [1,4]. Based on the hair growth rate, segmental hair analyses might demonstrate both long-term drug exposure as well as patterns of drug exposure [1].

One drug of interest, flunitrazepam (FLU), was the hypnotic benzodiazepine of choice among users of illegal drugs in Europe and the US for many years [5–10]. Rohypnol is one brand name of FLU, and this drug is not legally for sale in some countries nowadays [11]. The use and abuse of FLU has decreased, and FLU has been replaced by other benzodiazepines such as clonazepam in many Europe countries [7,8,12]. FLU is listed in the Catalog of Psychotropic Drugs and is controlled in China. Rohypnol is legally produced and prescribed for medical use in Japan. Rohypnol is readily available on the internet, with convenient delivery services and is smuggled into China through illegal internet shopping. This has allowed FLU to emerge as a rapidly growing substance in the clandestine market. FLU has become increasingly popular among young adults in Shanghai [13]. Hair analyses could show the trends and characteristics of FLU use, and this is imperative to allow establishment of suitable policy, treatment, prevention, and intervention measures regarding drug abuse. Hair segment analysis is recommended as it permits investigation of whether repeated use of the drug has occurred. To a certain degree, knowing the concentration of drugs in hair facilitates discrimination between a sporadic exposure and a chronic exposure. Hair analysis can detect doses of FLU as low as 1 mg. A likely limit of detection (LOD) required for FLU and the metabolite 7-aminoflunitrazepam (7AF) based on known data to detect single low doses was recommended to be lower than 0.0005 ng/mg [14,15]. Chronic users gave the FLU and 7AF concentrations of 0.02–3.9 ng/mg and 0.0088–9.5 ng/mg in hair, respectively [16–22].

Numerous studies [23–28] have been published regarding the method development and validation for the determination of FLU and the metabolite 7AF in hair. Nonetheless, only limited data are available regarding the concentrations of both FLU and 7AF in hair segments obtained from long-term FLU users [16]. Metabolite-to-drug concentration ratios of hair samples may represent a helpful tool for the differentiation of drug intake from external contamination [29]. There is a comparative lack of the reference metabolite-to-drug ratio values in FLU users' hair. To fill this knowledge gap, we aimed to investigate the concentrations of FLU and 7AF in a series of FLU users. Our objectives were to first develop and validate a method suitable for the determination of FLU and 7AF in drug users' hair and to compare concentrations in hair as well as parent/metabolite ratios to existing literature.

This study describes a method for using LC–MS/MS to simultaneously quantify FLU and 7AF in 1 cm hair segments from 22 FLU users. The results will expand the quantitative database. The purpose of this study was to provide concentrations of FLU and 7AF in hair segments of 22 FLU users and to give a comprehensive overview of quantitative data with regard to comparability.

## Materials and methods

### Reagents and chemicals

Reference standards, including FLU, 7AF, and the deuterated internal standards (IS) alprazolam-d_5_, were all bought from Cerilliant (Round Rock, TX, USA). Ammonium acetate (HPLC grade) was purchased from Honeywell Fluka^TM^ (Seelze, Germany). HPLC grade methanol and acetonitrile were obtained from Sigma-Aldrich (St. Louis, MO, USA). HPLC grade ammonium formate and formic acid were bought from CNW Technologies (Shanghai, China). Analytical grade acetone was obtained from Shanghai Lingfeng Chemical Reagent Co., Ltd. (Shanghai, China). Ultra-purified water was produced with a Milli-Q system (Millipore, MA, USA).

### Hair samples

Blank hair samples donated from healthy laboratory volunteers with written informed consent were used for quality control (QC) and calibrator preparation. The blank hair samples were washed and then dried overnight.

One QC hair sample was prepared by soaking blank hair samples (2–3 mm pieces) with a standard solution containing the analytes at 1.0 μg/mL in a 25:25:50 (v/v/v) mixture of methanol/acetonitrile/2 mmol/L ammonium formate (in 8% acetonitrile, pH 5.3) for 1 h with regular shaking. The hair was washed and dried overnight and stored at room temperature. QC sample was prepared as described by Nielsen and Johansen [30].

### LC–MS/MS

The detection was performed by a system consisted of an Acquity^TM^ Ultra Performance LC system (Waters Corporation, Milford, MA, USA) coupled to an API 4000 Q-TRAP mass spectrometer (Applied Biosystems/MSD Sciex, Foster City, CA, USA). Ionization was performed in positive mode. Separation was conducted on an Allure PFP propyl column (100 × 2.1 mm, 5 μm) from Restek (Bellefonte, PA, USA) at room temperature and a flow rate of 0.25 mL/min. The mobile phase was 20 mmol/L ammonium acetate containing 0.1% formic acid in water (A1) and acetonitrile (B1). Isocratic elution (30% A1 and 70% B1) was performed within 6 min. The optimal MS parameters were as follows: ion spray voltage, 5 500 V; source temperature, 450 °C; curtain gas, 25 psi; and nebulizing gas (GS1) and heater gas (GS2) were both 35 psi. Nitrogen was used as GS1, GS2, and the curtain gas, and the instrument was operated in multiple reaction monitoring (MRM) mode. Table 1 describes the parameters of each compound, including precursor ions, decluster potential (DP), product ions, collision energy (CE), retention times, and ion ratios. The retention time and the ion ratio of two MRM transitions were used to identify the analyte. The criteria were acceptable when the retention time and the ion ratio were ±2% and ±15%, respectively.

**Table 1.** Multiple reaction monitoring (MRM) parameters and retention times of analytes.

Compound	Precursor ion (*m/z*)	Product ion (*m/z*)	DP (V)	CE (eV)	RT (min)	Ion ratio
FLU	314.2	268.3	80	35	1.77	0.37
		239.3		45		
7AF	284.2	135.2	80	39	1.38	3.49
		226.2		41		
Alprazolam-d_5_	314.1	286.1	80	33	2.19	
		279.2		32		

DP: decluster potential; CE: collision energy; RT: retention time; FLU: flunitrazepam; 7AF: 7-aminoflunitrazepam.

Note: The first MRM transition for each analyte was used for quantification.

Data handling was performed by Analyst 1.4.2 software (Applied Biosystems).

### Hair sample preparation

Authentic hair strands were carefully aligned and cut into several segments of 1 cm length. Three to six sections were segmented depending on the hair length. Ten milligrams of the segmented hair were weighed into a plastic extraction tube (2 mL; Omni International, Inc., Kennesaw, GA, USA). Hair segments were rinsed sequentially with 1 mL acetone and twice with 0.5 mL water and shaken for 1 min prior to the analysis. The hair segments were then dried overnight at room temperature and ceramic beads were added.

FLU and 7AF were extracted from hair with an extraction medium consisting of a mixture of 25:25:50 (v/v/v) methanol/acetonitrile/2 mmol/L ammonium formate (in 8% acetonitrile, pH 5.3) as used by Nielsen et al. [31]. Briefly, 500 µL of the extraction medium, along with 50 µL of the IS (100 ng/mL), were added to a 2 mL extraction tube. The hair was then wet-milled with an Omni Bead Ruptor 24 coupled to an Omni BR-Cryo cooling unit (Omni International, Inc.). The speed was 6 m/s, with time 10 × 20 s and dwell time 10 × 40 s. After centrifugation for 3 min at 9 700 *g*, 5 μL of the supernatant was injected into the system. Duplicate determinations were used for the extraction of FLU and 7AF.

### Method validation

The method for the detection of FLU and 7AF in hair was validated according to Peters et al. [32] and the international guidelines [33]. The selectivity, LOD, limit of quantification (LOQ), linearity, accuracy, precision, extraction recovery (RE), matrix effect (ME), process efficiency (PE), and stability were examined.

The selectivity was evaluated by analyzing blank hair matrices collected from 10 different healthy individuals. These blank matrices were spiked with 100 common pharmaceuticals and drugs of abuse (as presented in Supplementary Table S1).

The calibration curves at 0.005, 0.01, 0.02, 0.05, 0.1, 0.2, 0.5, 2, 5, and 10 ng/mg were prepared by spiking six replicates of blank hair with different standard solutions prior to extraction. The calibration curves were derived by plotting the peak area of the analyte to IS versus the analyte concentrations in hair. The residual plots and the squared correlation coefficients (*r*^2^) were evaluated.

The LOD was evaluated by decreasing the analyte concentration until a signal-to-noise (S/N) ratio was higher than three. The LOQ was the lowest concentration yielding precision and accuracy within ±20%, which also fulfilled the requirement of an S/N ratio above ten.

The ME, RE, and PE were evaluated with six different blank hair samples at two concentration levels (1 and 5 ng/mg), as described by Matuszewski et al. [34]. For each level, the absolute peak area for each analyte obtained when spiked before extraction was designated as A; the absolute peak area for each analyte obtained when the same amount of analyte was spiked after extraction was designated as B; and the absolute peak area of each pure standard was designated as C. RE (%) = A/B × 100%, ME (%) = (B/C–1) × 100%, and PE (%) = C/A × 100%.

The precision and accuracy were determined using the blank hair of the analytes spiked at three levels (0.01, 0.5, and 5 ng/mg). The spiked samples were determined in four replicates at each level over four days (*n* = 16). The intermediate precision was evaluated by the determination of one QC measured in six series over 10 days. The criteria were considered acceptable when accuracy (bias%) and precision (relative standard deviation [RSD%]) were within ±20% of the LOQ and lower than 15% for other levels.

The stability of hair extracts stored in the autosampler was tested after 24 h.

### Authentic cases

In total, 22 authentic cases were analyzed. These cases were requests for hair analysis to detect the abuse of drugs and represented routine cases submitted by the police to the Academy of Forensic Science (Shanghai, China). All the flunitrazepam users signed the written informed consents for the analysis of their hair shafts in this study. The hair shafts were collected from the vertex posterior region of the head, as close to the scalp as possible, and were stored at room temperature until the analysis. Segmental hair analyses were performed on S1 (0–1 cm), S2 (1–2 cm), S3 (2–3 cm), S4 (3–4 cm), S5 (4–5 cm), and S6 (5–6 cm), where S1 was the proximal segment. Duplicate replicates were carried out for each segment.

## Results and discussion

### Method validation

The method was confirmed to be selective for the analytes because no interfering peaks were observed for the analytes in the ten blank hair samples or in the ten blank hair samples spiked with 100 common pharmaceuticals and drugs of abuse.

**Figure 1.**
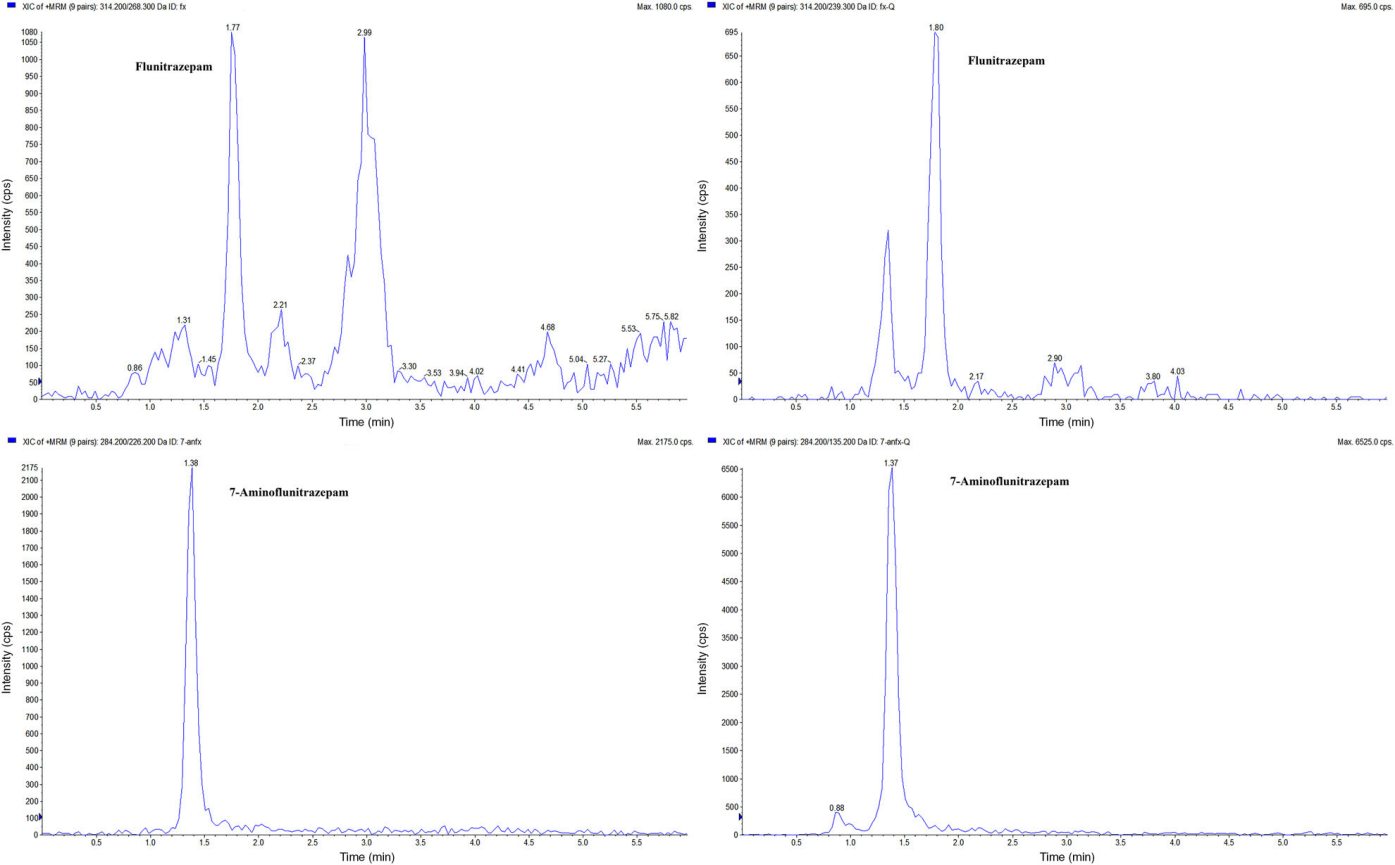
Multiple reaction monitoring (MRM) chromatograms of a hair segment from one authentic user displaying the concentrations of flunitrazepam and 7-aminoflunitrazepam (0.01 and 0.05 ng/mg, respectively).

The LOD and LOQ for FLU and 7AF were 0.005 and 0.01 ng/mg, respectively. The LOD, LOQ, and calibration parameters for FLU and 7AF are listed in Table 2. Linear regression with 1/*x*^2^ weighting was selected for the calibration curves, with acceptable correlation coefficients (*r*^2^ > 0.99). The LOQs for FLU and 7AF in this study are higher than in some previously published studies [15,19,35,36]. The published studies achieved LOQs below 0.005 ng/mg. In Negrusz et al. [31], the LOQ was 0.0025 ng/mg for FLU and 0.0005 ng/mg for 7AF using NCI-GC-MS to detect single doses, 10 times lower than our method. The applied extraction procedures in the published studies were either liquid liquid extraction (LLE) or solid phase extraction (SPE), which were complicated and time consuming. In our study, hair was extracted with extraction medium utilizing cryogenic grinding, and no incubation and evaporation was involved. Our method is not applicable for DFC cases, where a single dose has to be detected and is utilized for identifying chronic FLU users. The sensitivity of this method was sufficient to determine FLU and the metabolites in hair from chronic users. MRM chromatograms of FLU and 7AF in a hair segment from one authentic user are shown in Figure 1. Moreover, the single-step extraction in this study was more rapid than other preparation procedures [14,15,17,18,36,37]. Less hair (10 mg *vs.* 50 mg) was used in this study compared to our previous method for the benzodiazepines detection in hair [26] or methods published by other researchers [17,18,38,39] (An exception is the study by Miyaguchi [19], who carried out a liquid–liquid extraction with acetonitrile from 5 mg of hair).

**Table 2.** Limit of detection (LOD), limit of quantification (LOQ), and calibration for analytes in human hair.

Compound	LOD (ng/mg)	LOQ (ng/mg)	Calibration range (ng/mg)	Calibration curve	*r*
FLU	0.005	0.01	0.01–10.00	*y* = 1.11248*x* + 0.00301	0.99797
7AF	0.005	0.01	0.01–10.00	*y* = 11.24089*x* + 0.14727	0.99902

FLU: flunitrazepam; 7AF: 7-aminoflunitrazepam.

The precision and accuracy data of FLU and 7AF are reported in Table 3. The accuracy (bias%) was within ±15%. The inter- and intra-precisions for the replicate analysis were below 15%. The accuracy and precision results fulfilled the requirements set forth in the guidelines. The intermediate precision of the QC samples was 16.7% and 11.4% for FLU and 7AF (Table 3), respectively, and the results showed satisfactory precision and accuracy.

**Table 3.** Accuracy and precision for analytes in human hair.

Compound	Concentration	Accuracy (%)	Precision (RSD%)		QC (*n* = 6)	
	(ng/mg)	(*n* = 4)	Intraday (*n* = 4)	Interday (*n* = 16)	Concentration (ng/mg)	RSD%
FLU	0.01	+8.9	3.6	10.4	0.24	16.7
	0.5	−3.1	2.5	4.2		
	5	−14.1	1.2	2.9		
7AF	0.01	−12.1	4.6	9.1	0.27	11.4
	0.5	−7.0	3.8	6.4		
	5	−11.6	6.9	3.8		

FLU: flunitrazepam; 7AF: 7-aminoflunitrazepam; QC: quality control; RSD%: relative standard deviation.

The results of the RE, ME, and PE are shown in Supplementary Table S2. The RSD% of ME was below 13% for the two analytes at both levels, indicating that the hair samples from different individuals resulted in reproducible ME.

The 7AF levels in hair extracts were stable when stored in the autosampler for 24 h, with accuracy of −9% to 18%. FLU appeared to slightly increase to 27% (accuracy of −17% to 27%).

### Concentrations in hair segments of flunitrazepam users

This study presents the results of FLU and 7AF in 22 flunitrazepam users. The subjects were 16 women (73%) and six men (27%) ranging in age from 20 to 48 years (median age 34 years). Young women, particularly in the age range of 20–30 years, predominated among these drug users. Ten subjects had dyed or bleached hair, as assessed by the colour difference along the hair shafts and discolouration of the wash solvent or extraction medium. The remaining 12 subjects had a natural black hair colour (Table 4). FLU and 7AF concentrations in the hair segments for each subject are reported in Table 4.

**Table 4.** The concentrations of drugs in each hair segments of users (ng/mg). The LODs for FLU and 7AF were 0.005 ng/mg.

No.	Age (years)	Gender	Hair colour	Hair length (cm)	Drug	S1	S2	S3	S4	S5	S6	Mean	Range
1	38	F	Black	35	FLU	ND	+	0.01	+	+	+	0.01	0.01
					7AF	0.03	0.04	0.01	ND	ND	ND	0.03	0.01–0.04
2	23	F	Black	42	FLU	+	+	+	+	+	+	+	+
					7AF	0.04	0.03	0.02	0.04	0.08	0.08	0.05	0.02–0.08
3	28	F	Black	22	FLU	0.03	0.03	0.02	0.02	0.01	+	0.02	0.01–0.03
					7AF	0.11	0.06	0.10	0.20	0.14	0.06	0.11	0.06–0.20
4	28	F	0–3 cm: black; 3–6 cm: brown (dyed)	47	FLU	0.06	0.06	0.04	0.03	0.02	+	0.04	0.02–0.06
					7AF	0.24	0.20	0.04	+	+	0.04	0.13	0.04–0.24
5	48	F	Black	14	FLU	0.09	0.07	0.05	0.05	0.02	0.01	0.05	0.01–0.09
					7AF	0.27	0.19	0.22	0.22	0.07	0.02	0.16	0.02–0.27
6	34	M	Black	7	FLU	0.02	0.03	0.03	0.02	0.02	0.02	0.02	0.02–0.03
					7AF	0.14	0.19	0.23	0.20	0.18	0.16	0.18	0.14–0.23
7	37	M	Black	3	FLU	+	+	+	/	/	/	+	+
					7AF	0.05	0.04	0.07	/	/	/	0.05	0.04–0.07
8	23	F	Brown (dyed)	26	FLU	ND	+	+	+	+	+	+	+
					7AF	0.03	0.05	0.04	0.02	0.02	0.01	0.03	0.01–0.05
9	33	M	Black	6	FLU	0.16	0.14	0.16	0.15	0.12	/	0.15	0.12–0.16
					7AF	0.34	0.28	0.21	0.17	0.12	/	0.02	0.12–0.34
10	24	F	Black	54	FLU	0.05	0.04	0.02	0.02	0.03	0.03	0.03	0.02–0.05
					7AF	0.10	0.01	0.05	0.04	0.05	0.07	0.05	0.01–0.10
11	30	F	Brown (dyed)	49	FLU	0.03	0.04	0.07	0.07	0.09	0.09	0.06	0.03–0.09
					7AF	0.25	0.32	0.24	0.34	0.30	0.29	0.29	0.24–0.34
12	30	M	Black	7	FLU	0.05	0.06	0.08	0.07	0.07	0.06	0.06	0.05–0.08
					7AF	0.06	0.09	0.06	0.08	0.05	0.04	0.06	0.04–0.09
13	29	F	0-1 cm: black; 1–6 cm: brown (dyed)	11	FLU	0.07	0.07	0.07	0.05	0.05	0.03	0.06	0.03–0.07
					7AF	0.17	0.13	0.11	0.14	0.19	0.20	0.16	0.11–0.20
14	33	M	Black	3	FLU	0.06	0.06	0.03	/	/	/	0.05	0.03–0.06
					7AF	0.32	0.19	0.16	/	/	/	0.22	0.16–0.32
15	32	F	Brown (dyed)	36	FLU	0.02	0.02	0.03	0.03	0.02	0.03	0.025	0.02–0.03
					7AF	0.04	0.07	0.14	0.17	0.16	0.19	0.13	0.04–0.19
16	21	F	0–4 cm: black; 4–6 cm: brown (dyed)	41	FLU	0.03	0.02	0.02	0.02	0.02	0.02	0.02	0.02–0.03
					7AF	0.01	0.01	0.02	0.04	0.06	0.09	0.03	0.01–0.09
17	30	F	Black	29	FLU	0.04	0.03	+	+	+	+	0.03	0.03–0.04
					7AF	0.08	0.04	+	ND	+	ND	0.06	0.04–0.08
18	26	F	Brown (dyed)	23	FLU	0.01	0.02	0.02	0.02	0.02	0.01	0.02	0.01–0.02
					7AF	+	+	+	+	0.01	+	0.01	0.01
19	21	F	0–2 cm: black; 2–6 cm: brown (dyed)	36	FLU	ND	ND	ND	+	+	ND	ND	+
					7AF	+	+	0.03	0.04	0.04	0.04	0.04	0.03–0.04
20	22	F	Brown (dyed)	42	FLU	0.02	ND	+	ND	ND	ND	0.02	0.02
					7AF	0.11	0.15	0.03	+	+	ND	0.10	0.03–0.15
21	23	M	Black	5	FLU	ND	ND	0.02	0.02	/	/	0.02	0.02
					7AF	0.03	0.04	0.12	0.13	/	/	0.08	0.03–0.13
22	27	F	0–1 cm: black; 1–6 cm: brown (dyed)	25	FLU	0.06	0.04	0.02	0.02	0.02	+	0.03	0.02–0.06
					7AF	0.18	0.18	0.23	0.27	0.22	0.07	0.19	0.07–0.27

LOD: limit of quantification; FLU: flunitrazepam; 7AF: 7-aminoflunitrazepam; S1: 0–1 cm; S2: 1–2 cm; S3: 2–3 cm; S4: 3–4 cm; S5: 4–5 cm; S6: 5–6 cm; S1–S6: Segment 1–Segment 6; /: No hair; +: <LOQ; ND: not detected; M: male; F: female.

The concentrations of FLU in the hair segments of 22 users ranged from 0.01 to 0.16 ng/mg, with a median of 0.03 ng/mg, and those of 7AF ranged from 0.01 to 0.34 ng/mg, with a median of 0.09 ng/mg. The results showed that the concentration of the 7-amino-metabolite was higher than that of its parent compound, which concurs with the results in previous publications [15,16,40,41]. The reason could be that the 7-amino-metabolite is more readily incorporated into hair than its parent drug, owing to the increased basicity of the 7-amino-metabolite.

Xiang et al. [40] and Dumestre-Toulet and Eysseric-Guérin [42] included FLU and 7AF when describing hair analysis in DFC case reports and controlled studies in their comparative work; however, to our knowledge, no comprehensive overview has yet been presented for FLU and 7AF in various type of cases. Table 4 presents a comprehensive overview of the quantitative data for FLU and 7AF in human hair. Unfortunately, most of the listed publications did not detail hair colour.

As shown in Table 5, a single dose of 1 mg of FLU to one volunteer resulted in a 7AF concentration of 0.0035 ng/mg [14], while a 2 mg dose (*n* = 10) produced concentrations of FLU at 0.0005–0.0023 ng/mg and 7AF at 0.0005–0.008 ng/mg [15]. In several DFC cases, the hair concentrations of FLU were barely detectable, while 7AF concentrations ranged from 0.0015–0.078 ng/mg (median 0.0052 ng/mg) [35–37,43,44]. Our results for FLU and 7AF were much higher than those reported in these controlled single-dose studies and in DFC cases [14,15,35–37,43–45]. The hair concentrations of FLU and 7AF in chronic users were 0.02–3.9 ng/mg and 0.0088–9.5 ng/mg, respectively [16–22]. The hair concentrations in our study therefore likely arose from repeated use. Moreover, FLU and 7AF were generally distributed along the hair segments in most cases, which might indicate the chronic use of FLU. However, the results with regard to the time period of long-term drug intake should be interpreted with caution [46]. Diffusion from sweat or sebum, variations in hair growth rate, and the inconsistent collection of hair might also cause a spread of drug concentrations in several consecutive segments.

**Table 5.** Overview of quantification data of flunitrazepam and the metabolite in human hair.

Type	*n*	Hair colour	Hair length (cm)	Dosage	Hair concentration (ng/mg)		Wash method	Extaction method	Analytical method	LOD (ng/mg)
					FLU	7AF				
Controlled studies	1	Not given	5-mm root hair segment	A single dose of 1 mg Rohypnol^®^	ND	0.0035	Not given	Not given	GC–MS	Not given [14]
	10	Brown (3), Red (1), Gray (3), Blonde (1), Black (2)	0–1.5 cm	A single dose of 2 mg Rohypnol^®^	<LOQ (0.0025)	<LOQ (0.0005)–0.008	None	Hydrolyzed SPE	GC–MS	FLU: 0.0005; 7AF: 0.0002 [15]
	5	Not given	0–2 cm	Dose unknown	ND	0.0005–0.005	Not given	Not given	LC–MS	Not given [45]
DFC	1	Black	0–3 cm; 3–6 cm	–	0–3cm: 0.018; 3–6 cm: ND	0–3cm: 0.02; 3–6 cm: 0.008	Methanol and water	Methanol	LC–MS/MS	0.001 [35]
	1	Not given	Unknown	–	<LOQ (0.002)	0.0042	DCM	LLE	LC–MS/MS	FLU: 0.001; 7AF: 0.002 [37]
	2	Not given	0–2 cm	–	ND	0.0015–0.003	DCM	LLE	LC–MS/MS	0.0005–0.002 [36]
	1	Not given	0–2 cm	–	ND	0.0052	DCM	LLE	LC–MS/MS	Not given [44]
	1	Not given	–	–	ND	0.0317	DCM	LLE	LC–MS/MS	Not given [44]
	1	Not given	0–3 cm	Repetitive administered by the wife	ND	0.078	Washed (solvent not given)	LLE	LC–MS/MS	Not given [43]
	1	Not given	0–3 cm; 3–6 cm; 6–10 cm	An offender in a DFSA	0–3 cm: 0.03; 3–6 cm: 0.03; 6–10 cm: 0.01	0–3 cm: 0.21; 3–6 cm: 0.29; 6–10 cm: 0.02	Methanol and water	Methanol	LC–MS/MS	Not given [48]
Occasional intake	3	Not given	–	–	ND	0.0042–0.052	Not given	Not given	–	Not given [45]
Chronic users	1	Black	0–2 cm	2 mg/day of Rohypnol^®^	0.046		Washed (solvent not given)	LLE	LC–MS	FLU: 0.001; 7AF: 0.004 [19]
	31	Not given	Unknown	–	0.019–0.148		DCM	LLE	GC–MS	FLU: 0.015 [20]
	8	Not given	0–3 cm	–	0.02–9.5		Warm water and acetone	SPE	GC–MS	7AF: 0.02 [17]
	1	Not given	0–3 cm; 3–6 cm; 6–10 cm	–	0–3 cm: 0.0895; 3–6 cm: 0.0778; 6–10 cm: 0.072	0–3 cm: 0.024; 3–6 cm: 0.0088; 6–10 cm: 0.0122	DCM	LLE	GC–MS	Not given [18]
	1	Not given	0–3 cm; 3–6 cm; 6–9 cm	5–8 mg/day Rohypnol^®^	0–3 cm: 3.9; 3–6 cm: 3.7; 6–9 cm: 3.2		DCM, water, and methanol	On-line extraction	HPLC–PDA	0.2 [21]
	6	Black	6 × 1 cm	1–30 mg/month	<LOQ (0.01)	0.01–0.05	Washed (solvent not given)	Methanol	LC–MS/MS	Not given [16]
	1	Not given	0–2 cm	–		0.09	Isopropanol, and phosphate buffer	SPE	GC–MS	F and 7AF: 0.025 [49]
Postmortem cases	1	Gray	–	Suicide using prescription drugs	0.00176	0.0486	None	Mehtanol, SPE	GC–MS	FLU: 0.0015; 7AF: 0.0002 [22]
	1	Bleached a few months before death	–	Suicide using prescription drugs	0.023	0.0261	None	Mehtanol, SPE	GC–MS	FLU: 0.0015; 7AF: 0.0002 [22]
	26	Not given	–	Drug addicts deceased from fatal heroin overdose	0.031–0.129	0.003–0.161	DCM	LLE	GC–MS	FLU: 0.015; 7AF: 0.003 [41]

FLU: flunitrazepam; 7AF: 7-aminoflunitrazepam; LOD: limit of quantification; DFC: drug facilitated crime; DCM: dichloromethane; DFSA: drug facilitated sexual assault; LLE: liquid liquid extraction; ND: not detected; SPE: solid phase extraction.

The present study is limited by the fact that the doses and ingestion periods of the FLU users can rarely be obtained. However, the hair concentrations of FLU and 7AF from chronic users are still lacking. Therefore, our concentration values of both drugs are valuable for forensic toxicology purposes.

Metabolite to drug ratios might be useful for the interpretation of the results [29]. Therefore, the ratio of 7AF to FLU in hair segments was analyzed. The median ratios of 7AF to FLU were 2.75, 2.43, 2.66, 3.68, 3.49, and 3.07, respectively, for each segment (S1 S2, S3, S4, S5, and S6), with ratios of the 10th–90th percentiles of 0.51–8.29 for all hair segments (median value 3.07). Similarly, a median 7AF to FLU ratio of 3.13 has been reported in the hair of three postmortem cases [47]. Metabolite-to-drug concentration ratios in FLU users’ hair might provide useful information on drug metabolism and subsequent hair analysis.

## Conclusion

The LC–MS/MS method developed for the determination of FLU and 7AF in hair samples is simple, rapid, and robust. This manuscript presents the concentrations of FLU and 7AF determined by segmental analysis of up to six 1 cm hair segments from 22 flunitrazepam users. The concentrations of FLU and 7AF ranged from 0.01 to 0.16 ng/mg and 0.01 to 0.34 ng/mg, respectively. Most cases had concentrations of FLU and 7AF that were suggestive of repeated drug use. A summary of the published concentrations gives valuable data and can assist forensic investigators in their estimations of drug use history and patterns. Even though the interpretation of hair concentrations cannot be easily compared, this knowledge can be crucial for improving the interpretation of the results. The metabolite-to-drug ratio values in the hair reported in the present study may be used as reference values to confirm FLU intake because the values were from FLU users.

## Supplementary Material

Supplemental Material
