# Supplementary material for: Segmental hair analysis for flunitrazepam and 7-aminoflunitrazepam in users: a comparison to existing literature
Source: Forensic Sci Res. 2020 Oct 22;7(2):299–307. doi: 10.1080/20961790.2020.1824600 (PMC9245979; doi:10.1080/20961790.2020.1824600)
Supplement: Supplemental Material [file TFSR_A_1824600_SM3576.docx]

Table A.1. List of potential interfering compounds in the selectivity experiment.

| Potential interfering compounds | | | | |
| --- | --- | --- | --- | --- |
| 7-Aminoclonazepam | 9-OH-Risperidone | α-OH-Alprazolam | α-OH-Midazolam | α-OH-Triazolam |
| Alprazolam | Aminopyrine | Amisulpride | Antipyrine | Aripiprazole |
| Aspirin | Atropine | Azithromycin | Benazepril | Benzhexol |
| Bupivacaine | Buspirone | Canapril | Carbetapentane | Cefradine |
| Chlordiazepoxide | Chloroquine | Chlorphenamine | Chlorpromazine | Cinnarizine |
| Citalopram | Clenbuterol | Clonazepam | Clozapine | Dehydroaripiprazole |
| Desmethylclozapine | Dexmedetomidine | Dextromethorphan | Dezocine | Digitoxin |
| Diphenhydramine | Diphenoxylate | Ethambutol | Etomidate | Famotidine |
| Fenfluramine | Fosinopril | Glibenclamide | Gliclazide | Haloperidol |
| Ibuprofen | Indometacin | Irbesartan | Isoniazid | Lamivudine |
| Lidocaine | Lincomycin | Lorazepam | Metformin | Methaqualone |
| Metoclopramide | Midazolam | Mirtazapine | Naloxone | Naproxen |
| Nefopam | Neostigmine | Nimetazepam | Norchlordiazepoxide | Nordiazepam |
| Norolanzapine | Norsertraline | Olanzapine | Omeprazole | Oxazepam |
| Paracetamol | Parecoxib | Paroxetine | Penehyclidine | Perphenazine |
| Phenacetin | Phenformin | Phentermine | Procaine | Propofol |
| Propoxyphene | Pyrazinamide | Ramosetron | Rifampicin | Risperidone |
| Salbutamol | Salicylic acid | Secobarbital | Sertraline | Sildenafil |
| Sulpiride | Temazepam | Topiramate | Tramadol | Trazodone |
| Triazolam | Trifluoperazine | Trifluoroacetic acid | Zolpidem | Zopiclone |

Note: All potential interfering compounds were spiked at 1.0 ng/mg in hair.

Table A.2. Recovery, matrix effect, and process efficiency for analytes in human hair.

| Compound | Concentration | ME (%) |  | RE (%) | PE (%) |
| --- | --- | --- | --- | --- | --- |
|  | (ng/mg) | (n=6) | RSD % | (n=6) | (n=6) |
| Flunitrazepam | 1 | -16 | 3 | 95 | 81 |
|  | 5 | -13 | 5 | 91 | 80 |
| 7-Aminoflunitrazepam | 1 | -54 | 12 | 92 | 43 |
|  | 5 | -53 | 13 | 86 | 40 |
